# Cardiac rehabilitation program: An exploration of patient experiences and perspectives on program dropout

**DOI:** 10.1111/wvn.12554

**Published:** 2022-01-17

**Authors:** Monica Lee, Timothy Wood, Sammy Chan, Elsa Marziali, Tricia Tang, Davina Banner, Scott A. Lear

**Affiliations:** ^1^ 1763 Department of Biomedical Physiology and Kinesiology Simon Fraser University Burnaby British Columbia Canada; ^2^ 6727 School of Nursing University of Northern British Columbia Prince George British Columbia Canada; ^3^ Department of Medicine University of British Columbia Vancouver British Columbia Canada; ^4^ Rotman Research Institute University of Toronto Toronto Ontario Canada; ^5^ 1763 Faculty of Health Sciences Simon Fraser University Burnaby British Columbia Canada

**Keywords:** cardiac rehabilitation, cardiovascular disease, patient experience, patient preference, program dropout, qualitative, rehabilitation

## Abstract

**Background:**

Cardiac rehabilitation programs (CRP) are effective evidence‐based secondary prevention programs that reduce morbidity and mortality in patients with cardiovascular disease (CVD). However, participation remains suboptimal, resulting in under‐treatment and greater risk for recurrent cardiac events. Understanding the reasons behind CRP dropout is urgently needed to inform the development of programs that best meet patient needs and support sustained engagement.

**Aims:**

The aim of this study was to identify and understand factors impacting CRP dropout from the patient perspective.

**Methods:**

A qualitative study using semi‐structured interviews was undertaken to examine the experience of 23 patients who dropped out of a CRP within a large urban hospital in British Columbia, Canada. Data were coded, analyzed using the constant comparison technique, and organized thematically.

**Results:**

Participants described multiple challenges when attempting to complete CRP. Analysis of the data led to the identification of three main categories: (1) challenges living with CVD, (2) perceived advantages and disadvantages of CRP, and (3) unmet needs during CRP.

**Linking evidence to action:**

In the practice setting, assessment of readiness to engage in CRP, alongside patient preferences and engagement needs, should be undertaken for maximum CRP uptake and completion. Providing diverse modes of CRP delivery, along with exploring the impact of virtual options as compared to traditional in‐person programs, will further advance the CRP evidence and may help address pervasive access barriers.

## INTRODUCTION

Cardiac rehabilitation programs (CRP) are evidence‐based programs that seek to address the growing global burden of cardiovascular disease (CVD). The programs are proven to reduce ‐the risk of all‐cause hospital admissions, improve long‐term all‐cause mortality, and may improve health‐related quality of life (Long et al., 2019). As a result, the European Society of Cardiology has recognized cardiac rehabilitation as a class 1A recommendation in the care of patients with CVD (Piepoli et al., 2016), and there continues to be strong evidence to support its use in patients with CVD. Canada has developed detailed guidelines on the delivery of CRPs (Stone & Arthur, 2005), although guidelines, position statements, and policy documents have been developed globally (Price et al., 2016). Despite the strong evidence supporting the benefits of CRP, participation rates are less than 50% in most countries, with dropout rates as high as 82% (Bäck et al., 2017; Turk‐Adawi & Grace, 2015). This is a growing concern given that patients who prematurely discontinue CRP are consequently at greater risk of recurrent cardiac events or death (Pardaens et al., 2017).

Previous research has focused and reported on quantitative outcomes involving program and patient‐based factors that contribute to CRP non‐adherence (Turk‐Adawi & Grace, 2015). For instance, limited availability of CRP, lack of physician referral, financial constraints, distance, and transportation problems have been reported as common program‐based factors (Murray et al., 2012). Frequently reported patient‐related factors include older age, smoking, sex, low socioeconomic status, time conflicts, disinterest, and presence of co‐morbidities, such as diabetes (Grace et al., 2014). Despite these well‐known factors, less is known about CRP dropout from the perspective of the patient. For this paper, dropout is defined as those participants who completed more than one but less than half (18/32 sessions) of the program and were formally discharged.

Reasons for CRP dropout are multifaceted but may include patient psychological challenges like adjustment difficulties and distress following a cardiac event (Campkin et al., 2017). Some patients have reported undergoing a grieving process after a cardiac event due to the loss of health, self‐image, role function, and lifestyle, all of which can contribute to non‐adherence in CRP (Higgins et al., 2007; Jokar et al., 2017; Murray et al., 2012). Qualitative inquiry can facilitate a greater understanding of CRP patient experiences (Schopfer et al., 2016), including reasons for program dropout. A deeper understanding of the experiences of those who drop out of CRP is crucial to identify and develop successful program and policy initiatives that optimize CRP uptake and completion. Thus, the goal of this study was to identify and understand factors impacting CRP dropout from the patient perspective.

## METHODS

Qualitative studies are best suited to examining phenomena about which little is known (Morse, 1994). A qualitative approach was selected, as this allows for the examination of patient experiences and perspectives, including decision‐making and factors related to CRP dropout. The research adopted inductive and deductive approaches to analysis, using constant comparison to examine similarities and differences across the data and thematic analysis techniques to organize the emerging data. Qualitative research is grounded in the naturalistic context and values diverse experiences and perspectives, thus suiting the purpose of this inquiry (Morse, 1994).

### Participant recruitment

A purposive sample of patients who dropped out of an in‐person CRP offered at a large urban hospital in British Columbia, Canada, was selected. The CRP was a traditional multidisciplinary 4‐month intervention program supervised by cardiologists, nurses, dieticians, and American College of Sports Medicine certified exercise specialists and exercise leaders. The monitored exercise program consisted of aerobic exercise and resistance training 2 days per week for 16 weeks (32 sessions total). Exercise included warm‐up, aerobic activity with prescribed target heart rates determined by an exercise stress test, resistance training, and cooldown. Education was offered in groups and focused on secondary prevention of CVD, including management of risk factors.

Charts of patients enrolled in the CRP from January 2011 to March 2013 were identified (*n* = 993). Of the 993 individuals, 571 (58%) were excluded as they had either completed the CRP (*n* = 503, 51%), or the medical charts were inaccessible (*n* = 68, 7%). From this, 422 (42%) patients who did not complete the CRP were identified. Dropout was defined as individuals who had attended a minimum of two sessions and were formally discharged from the program. Among these participants, only individuals who signed the Cardiac Rehabilitation and Prevention Clinic Consent form consenting to contact for future research were screened for the study inclusion criteria (*n* = 339, 80%) and sent an invitation letter for the study. Two weeks following the mailing date, the research assistant followed up with a telephone call to those patients who had not responded to discuss the study.

A total of 248 (74%) declined participation, and five (1%) were hoping to return to the CRP and were thus excluded. Thirty‐five individuals (10%) were considered to be “unable to be reached” after being called five times over a 4‐week period, whereas 13 (4%) were hospitalized and 11 (3%) passed away before contact could be made. An additional four patients (1%) consented but did not complete the study. In total, 23 (7%) of the original 339 individuals participated in the study and completed an interview. The study underwent provincial harmonized research ethics board review (H11‐00515), and all participants provided informed consent.

### Data collection and analysis

In‐person, semi‐structured interviews were undertaken by a trained researcher with 23 participants who had dropped out of their CRP. The interview schedule was developed in consultation with clinical and qualitative research expertise team members. Questions were designed to explore the experiences of CRP, as well as decision‐making and factors related to CRP dropout. Examples of interview questions included “Can you tell me what it has been like for you to manage your heart condition since you were first diagnosed?” and “What role do you think CRP has in managing CVD?” The interview schedule was used to guide interviews, although participants were free to speak about their experiences. Participants were asked about their experiences of CRP, reasons for program discontinuation, positive and negative attributes of CRP, and challenges faced. Each interview lasted approximately 30 minutes and was audio‐recorded and transcribed verbatim.

Data were coded and constantly compared to generate rich interpretations of the data. First, data were coded by the main researcher, and important words, phases, and topics were identified. Through constant comparison, focused coding was undertaken to extract and refine the emerging data. Relationships within the coded data were explored for similarities and differences, leading to the identification of three themes. Data saturation, or the point at which no further information emerges from the data (Glaser & Strauss, 1967), was achieved.

To promote rigor, the emerging data were compared independently by two team members, and rich descriptions of the emerging data were created. Researchers further engaged in reflexive practices, including the creation of memos, to allow for self‐reflection, critical examination of interpretations, and to create an audit trail. These approaches were enlisted to promote transparency and openness of the analytical process.

## RESULTS

Participant characteristics are shown in Table 1. Participants had a mean age of 60.6 years (±14.5 years), the majority of which were men (65%, *n* = 15). No differences were found for the baseline characteristics between men and women, education, income, marital status, and smoking status. The number of completed CRP sessions ranged from two to 18 classes, with the median completed sessions being seven. Three categories were identified during analysis: (1) challenges living with cardiovascular disease, (2) perceived advantages and disadvantages to CRP, and (3) unmet needs from CRP.

**TABLE 1:** Sociodemographic characteristics of individuals who did not complete cardiac rehabilitation program

Demographics	Did not complete cardiac rehabilitation program (*N* = 23)
Male	15 (65%)
Age in years (mean [range])	60.6 ± 14.5
Completed sessions (mean [range])	7 (2.18)
Education	
High school	4 (17%)
College/diploma	5 (22%)
University/masters/PhD	14 (61%)
Income (dollars)	
N/A	3 (13%)
<30,000	6 (26%)
30,000–40,000	2 (9%)
40,000–50,000	0 (0%)
50,000–60,000	0 (0%)
>60,000	12 (52%)
Marital status	
Single	5 (21%)
Married	11 (48%)
Divorced	2 (9%)
Widowed	3 (13%)
Common law	2 (9%)
Smoking status	
N/A	0 (0%)
Never	8 (35%)
Former	13 (57%)
Current	2 (9%)

### Challenges living with cardiovascular disease

Participants explained how physical ailments or limitations associated with CVD made it difficult to adhere to CRP. Furthermore, there was a reciprocal effect of physical factors on participant mental well‐being. For some, the stress of coping with new physical limitations led to feelings of fear and anxiety regarding future health status. Thus, health‐related factors affecting CRP adherence were categorized into physical limitations and fear and uncertainty of future health.

#### Physical limitations

Adjusting to new physical limitations following a cardiac event was reported to be a major struggle for most of the participants, which they perceived as restricting their ability to participate in CRP. Feeling “out of breath,” “tired,” and “unable to move around” were common complaints. These new physical limitations were seen to compromise participant ability to engage in regular daily activities, including work, household chores, and grocery shopping. Participants stated that these limitations made it difficult to make necessary lifestyle changes to improve their health, including engaging in more frequent exercise. These physical hindrances directly contributed to the early discontinuation of CRP for several participants. For example, participant 34 commented “I was having physical problems at the time, and that's why I stopped coming [to CRP].” For others, these limitations made it challenging to balance and complete their usual activities alongside new healthy lifestyle expectations following their cardiac event. As participant 24 explained, “I stopped coming because I could not do everything.”

#### Fear and uncertainty of future health

Participants explained how the potentially life‐threatening nature of their cardiac event led to ongoing fears about the future and of dying. These fears often left participants feeling helpless and vulnerable to uncertainty, leading them to avoid situations that involved leaving their home or engaging in physical activity. For example, participant 21 stated, “I don't want to be walking down the street and keel over… I don't want that to happen again.” Fears of death or repeated cardiac events forced these participants to withdraw from many activities outside of the home.

During the interviews, participants also described how they were faced with the need to manage their negative emotions on a daily basis and that this made committing to CRP a challenge. Of note, anxiety and fear about their ability to undertake the physical activity as part of the CRP were commonly mentioned and deterred some from attending CRP. Despite this, many participants who dropped out of CRP commented about their longing to get “back to normal.”

### Perceived advantages and disadvantages of cardiac rehabilitation programs

Negative and positive attributes of CRP were described by participants. Factors relating to program characteristics were identified, and these were categorized into four categories: (1) motivation and support, (2) structure and routine, (3) time commitment and geographic separation, and (4) lack of a personalized exercise program.

#### Motivation and support

Despite early discontinuation, CRP was viewed by participants as important to recovery following a cardiac event and described as “motivational” and “encouraging.” Participant 15 explained “The [healthcare professionals] who run [CRP] motivate you… and they're interested in you.” Participants especially valued the psychosocial aspects of the program, including peer support. For many, the group setting was noted as being a positive attribute of the program as it enabled them to interact with healthcare professionals and other cardiac patients. As a result of these relationships and peer support, participants reported feeling comfort and assurance to engage in healthier activities to better manage their health. Participant 41 described “One of the difficulties that I had was exercising on my own… [but CRP] was social and it was actually enjoyable, the whole process.”

#### Structure and routine

Participants attributed value to CRP because it provided structure and routine. Through CRP, participants felt as though they were able to begin the process of rehabilitation and “become healthy again,” and that regular exercise set them “on the right path.” Participant 23 stated “It was nice to see people and be able to talk with them and it felt more like I was going to say a gym class instead of it being more like this thing because you're sick.” For others, CRP enabled them to re‐envisage their own future and ability to lead a normal life. Put by participant 12, “I think the most important thing [about CRP] is it makes us aware that we have heart disease and that we can go on living a very normal life with limitations.”

#### Time commitment and geographic separation

Participants also highlighted some key program‐related barriers and disadvantages that impacted their ability to complete the program. Time commitment was identified as a key reason for CRP dropout. Many participants reported they “had no time” for CRP or were bound by other work or family obligations. Other commonly reported issues included the location of the CRP and travel needs. For many, the distance to the program was found to be a contributing factor to early CRP discontinuation despite perceived benefits. Participant 15 explained, “I did [CRP] for 2 months… It was interesting to talk to people, like the trainers… but it did become quite inconvenient to be coming out here.” Overall, the accessibility of the program was a key factor in program participation and completion.

#### Lack of a personalized exercise program

Many participants also expressed dislike regarding the one‐size‐fits‐all nature of the exercise component of CRP. Dissatisfaction with the lack of individualized components of the exercise segment included concerns about exercise intensity. For example, participants who were previously physically active reported feeling “held back” in a group setting and did not feel challenged with the exercise regimen. Participants commented that “I get much better cardio workouts on my own” (participant 7) and “I thought that was rather slow to move me… up to another level” (participant 31). These participants felt the exercise training sessions could have been greater in intensity and shorter in duration, particularly when it came to cooldown and warm‐up. The ability to tailor the exercise sessions, reflecting the varied abilities and needs of participants, was seen as one way to overcome this barrier. Participant 36 explained “I think if you got people of similar levels at the same time, it would help.”

### Unmet needs during cardiac rehabilitation

#### Program accessibility and flexibility

When thinking about their experiences of CRP and decision to discontinue, participants explained that having more accessible and flexible community‐based CRPs was needed. In particular, having CRPs that are located outside the hospital setting and closer to their home location was important. Participant 23 suggested “I think a lot more centers, like community centers,… like the YMCA, if they could help get people in and… have programs like this… then I think more people would stick with it.” Overwhelmingly, participants noted that having more geographically accessible programs would support them to complete the program. For others, greater accessibility would also “make it easier to exercise” and that having a local exercise partner outside of CRP would greatly motivate them to exercise.

#### Additional time with providers

Participants also felt additional time with healthcare providers would be a form of support that could be beneficial. Some participants indicated their desire for more “face time” with the cardiologist. Participants identified that having more frequent contact with their cardiologist, as well as longer appointments, would be most helpful in assisting them to managing their CVD. Put by participant 24, “I mean [the doctor] is nice and everything… but it's just that there's not much interaction after months, you know?” Overall, participants reported that having ongoing rapport with the physician would ultimately help them feel more supported with their disease state and prognosis, thus encouraging program completion.

## DISCUSSION

Although barriers and facilitators of CRP uptake have been widely described in the literature (Dunlay et al., 2009; Grace et al., 2009; McDonall et al., 2013), fewer studies have explored reasons for CRP dropout from the patient perspective. In line with calls for more research to be conducted examining factors not easily measured quantitatively (Resurrección et al., 2019), this study explores patient experiences and perspectives of CRP dropout.

Participants in this study reported that the CRP was advantageous as it provided motivation, routine, and structure. Even among the participants who reported dissatisfaction with the exercise component, it was reported that the psychosocial aspects were valuable and reminded them that they were “not alone.” Having other cardiac patients present during CRP helped participants feel supported and assured regarding their disease. Other research has highlighted the importance of group settings for helping those with CVD deal with life stressors and gain stability (Carron & Prapavessis, 1997; Hinsz & Nickell, 2004). However, it is possible that the perceived benefits of CRP that were described may not be sufficient to ensure program completion when considering the complexity and interrelatedness of reasons for dropout.

Surviving a cardiac event is a traumatic experience for many patients, and undertaking major lifestyle changes was perceived as overwhelming and difficult for many of the participants. Most notably, patients had to adjust to new physical limitations (Duncan & Pozehl, 2003; Pfaeffli et al., 2012). The impact of these physical impairments all contributed toward CRP dropout in our sample. Anxiety surrounding CVD recurrence or progression was commonly reported, and this led to concern about being in public and participating in CRP. Several other studies have demonstrated similar findings (Carney et al., 2003; Clark et al., 2013; Glazer et al., 2002). In a study evaluating 46 patients who had participated in a CRP, the authors linked psychological functioning and program adherence, identifying depression as a predictor of program dropout (Glazer et al., 2002). Considerations related to the timing of CRP, along with patient readiness to engage in secondary prevention interventions, may be valuable and may optimize program uptake and completion.

Other factors that gave rise to CRP dropout included distance, time conflicts, and lack of a personalized exercise regime. For example, participants in this study found it challenging to attend CRP at scheduled times, either because of existing work or family commitments or as a result of lengthy commutes and travel. This is consistent with previous studies (Beswick et al., 2005; Herber et al., 2017; Turk‐Adawi & Grace, 2015). For example, in a qualitative examination of CRP nonattendance (Herber et al., 2017), authors described how several participants may not have attended the CRP had the location been less convenient or accessible. In response to these issues, the application of technology may provide opportunities to encourage ongoing CRP participation. For example, home‐based CRPs delivered using technology and the internet have been increasingly introduced to widen access and participation (Arthur et al., 2013; Lear et al., 2014; Leon et al., 2005). These home‐based CRPs have demonstrated success and cost‐effectiveness, illustrating their potential role in secondary prevention for patients who drop out of traditional CRPs (Arthur et al., 2013; Lear et al., 2014; Leon et al., 2005) and offering an appropriate and convenient alternative to in‐person CRPs (Banner et al., 2019). Such programs may minimize issues of program nonattendance related to distance, time constraints, and dissatisfaction with the exercise component (Bäck et al., 2017; Ragupathi et al., 2017). Supplementary community‐based CRPs offering varied exercise intensities targeting those suffering physical or psychosocial issues may enhance program completion.

Peer support from other cardiac patients during CRP was consistently noted as a key factor influencing CRP attendance. Thus, an important topic for future research is identifying the best sources of social support for cardiac patients with extensive physical or psychosocial vulnerabilities. Programs that are inclusive of partners or family members may be more efficacious than current individual‐oriented interventions (Rowland et al., 2017). Other key supports included having regular contact with healthcare providers, as was identified in our sample as an unmet need of participants. Existing research has illustrated that physician involvement has been associated with improved patient outcomes and long‐term medication adherence (Kulkarni et al., 2006; Rice & Lumsden, 2006). A previous study also found that the presence of a trusted physiotherapist helped instill motivation and support among patients who did not choose to attend CRP, demonstrating the importance of continuity of care and interdisciplinary supports beyond the hospital setting component (Bäck et al., 2017).

This study has identified that reasons for dropout are complex and reflect a broad range of intersecting physical, psychological, and psychosocial factors. Examining an individual's motivation and readiness to undertake CRP, along with the identification of potential barriers prior to CRP initiation, may prove valuable and may optimize CRP effectiveness. Furthermore, promoting diverse access points for CRP and providing options that best meet patient needs may minimize CRP dropout. This may include options for blended CRPs that incorporate both virtual and in‐person elements or may include programs that span different times and locations. However, further research is needed to examine the interplay of psychological well‐being, new physical impairments, and social support as they relate to program‐based characteristics. Further, considering that higher socioeconomic status seems to be associated with higher dropout rates, it may be worth investigating in future research.

### Strengths and limitations

This study has contributed new and enhanced understanding of factors that impact CRP completion and dropout. The study engaged patients undertaking CRP from a large urban center offering tertiary‐level care for persons with CVD, and our study sample allowed for data saturation to be achieved. Despite the many strengths, some limitations exist. First, study participants were recruited from a single urban center. Recruiting patients from multiple sites, including those accessing CRP in both urban and rural settings, may have yielded new and different insights. Second, our study sample included a small proportion of women. Greater diversity within the study sample, along with attention to other key demographic factors, such as ethnicity, may yield additional perspectives. Third, a large number of potential participants declined to participate. However, this was expected as patients who drop out of a program are difficult to reach and recruit. Finally, the interviews were conducted up to 2 years following dropout from the program; as such, it is possible that participants may have failed to recall some aspects of their experience.

## LINKING EVIDENCE TO ACTION

- In the practice setting, assessment of readiness to engage in CRP, alongside patient preferences and engagement needs, should be undertaken to maximum CRP uptake and completion.
- Providing diverse modes of CRP delivery, along with exploring the impact of virtual options as compared to traditional in‐person programs, will further advance the CRP evidence and may help address pervasive access barriers.
- As such, four recommendations are proposed: (1) Assess patient readiness to be engaged in CRP, (2) Assess patient preferences and engagement needs, (3) Provide diverse modes of CRP delivery, and (4) Consider virtual options for CRP delivery.

## CONCLUSIONS

Following an acute event, persons living with CVD face a wide range of health challenges including the need for ongoing monitoring and management as well as CRP to support healthy lifestyle practices and reduce the likelihood of recurrent cardiac events (Taylor et al., 2004; Wenger, 2008). This study sought to examine perspectives and factors related to CRP dropout. Analysis of the study data revealed that CRP dropout occurred as a result of a complex interplay of physical, emotional, and psychosocial factors, like anxiety and physical limitations, along with program‐based barriers related to accessibility, geography, and program responsiveness. Our findings reinforce the idea that accessible CRPs, along with attention to participant needs and readiness, are needed to optimize program uptake and completion. Leveraging the beneficial components of CRP though also considering the opportunity to utilize community‐based resources and technology may promote flexible ways to enhance adherence, further increasing the success and long‐term benefits of rehabilitative programs for this high‐risk population.
